# Real-Time Gait Cycle Parameter Recognition Using a Wearable Accelerometry System

**DOI:** 10.3390/s110807314

**Published:** 2011-07-25

**Authors:** Che-Chang Yang, Yeh-Liang Hsu, Kao-Shang Shih, Jun-Ming Lu

**Affiliations:** 1 Department of Mechanical Engineering, Yuan Ze University, 135 Yuan-Tung Rd., Chung-Li, Tao-Yuan 320, Taiwan; E-Mail: ccyang@saturn.yzu.edu.tw; 2 Division of Orthopedics, Shin Kong Wu Ho-Su Memorial Hospital, 95 Wen Chang Rd., Shih Lin District, Taipei 111, Taiwan; E-Mail: scorelin@ms28.hinet.net; 3 Gerontechnology Research Center, Yuan Ze University, 135 Yuan-Tung Rd., Chung-Li 320, Taiwan; E-Mail: jmlu@saturn.yzu.edu.tw

**Keywords:** accelerometry, accelerometer, Parkinson’s disease, gait, mobility

## Abstract

This paper presents the development of a wearable accelerometry system for real-time gait cycle parameter recognition. Using a tri-axial accelerometer, the wearable motion detector is a single waist-mounted device to measure trunk accelerations during walking. Several gait cycle parameters, including cadence, step regularity, stride regularity and step symmetry can be estimated in real-time by using autocorrelation procedure. For validation purposes, five Parkinson’s disease (PD) patients and five young healthy adults were recruited in an experiment. The gait cycle parameters among the two subject groups of different mobility can be quantified and distinguished by the system. Practical considerations and limitations for implementing the autocorrelation procedure in such a real-time system are also discussed. This study can be extended to the future attempts in real-time detection of disabling gaits, such as festinating or freezing of gait in PD patients. Ambulatory rehabilitation, gait assessment and personal telecare for people with gait disorders are also possible applications.

## 1. Introduction

Gait dynamics reflect one’s mobility which can be affected by physical impairment, age progress and changes in health status. Ambulatory gait parameters can be important measures to assess functional ability, balance control and to predict fall risk. Individuals with degenerative mobility, e.g., Parkinson’s disease (PD) patients or older adults usually have gait disorders such as reduced walking speeds with increased cadences, reduced step/stride lengths, and increased inter-stride variability [1]. PD patients of advanced stage might have encountered episodic gait disturbances, like festinating or even freezing of gaits (FOG) that could lead to falling and adverse health outcomes [2,3]. Regularity, rhythm and symmetry are important gait cycle parameters that can be apparently altered in walking patterns among people of varied mobility [2–4]. Therefore, the monitoring of the above gait cycle parameters can be beneficial to assess the mobility and risk of occurrence of episodic gait disturbances.

Gait evaluation is frequently based on observational interpretations which are subjective and may vary among clinicians or investigators. As a consequence, gait monitoring and analysis techniques have been widely developed and studied. Gait dynamics can be accurately measured by using optical motion capture systems which utilize high-speed infrared cameras to record the three-dimensional positions of retro-reflective markers attached to the joints and segments of the human body [5]. Gait detection techniques utilizing pressure sensors embedded in an overground walkway [6] have also been used. These techniques can detect foot contact (heel strike and toe-off) and even the foot pressure distribution to investigate temporal gait parameters. However, those systems are expensive, and the sophisticated instrumentation requires specialized personnel. Therefore the uses of those systems are usually limited in laboratory or clinical environments. Simpler systems based on pressure detection, such as the portable in-shoe pressure measurement system have also been presented [7,8]. The systems utilizing in-shoe pressure detection can only provide simple temporal gait measures while accelerometer-based or video-based systems can provide temporal and spatial gait measures, even accurate measurement of lower limbs and body movement.

Accelerometry using wearable systems has drawn a vast amount of research interest in the study of human movement. Accelerometers have widely been used in wearable systems for movement classification, fall detection, estimation of energy expenditure and gait analysis [9,10]. Accelerometers in combination with gyroscopes that measure angular velocity and accurate orientation have also been developed [11,12]. Though the pathological gaits have been well studied and described, only a few studies have investigated recognition of abnormal gaits using wearable accelerometry systems. A shank-mounted accelerometer was used to monitor the FOG in PD patients by means of a “freeze index” computed by frequency spectral analysis. However, the power spectral analysis can hardly be performed in real-time on compact wearable systems [13]. A wearable system using ARM7 processor was also demonstrated to detect FOG in real-time from every collected 0.32 s acceleration data [14]. Due to the computation constraints, it was reported that a longer sample data will produce longer latency of the system which might not be acceptable for practical uses.

The development of cost-effective approaches to real-time gait monitoring is important and beneficial. This paper presents the development of a wearable accelerometry system for real-time gait cycle parameter recognition. A waist-mounted wearable motion detector was designed to measure trunk accelerations during walking. The autocorrelation procedure is implemented in the system to derive several gait cycle parameters, including cadence, step regularity, stride regularly and step symmetry. For validation purposes, five PD patients and five young healthy adults were recruited in order to investigate whether the gait cycle parameters among the two subject groups of different mobility can be quantified and distinguished by the proposed system. Practical considerations and limitations for implementing real-time gait cycle parameter recognition in an embedded wearable system are also discussed. If the gait cycle parameters can be identified in real time, continuous detection of gait variability would be possible. This paper can lead to a future development of wearable systems enabling real-time recognition of abnormal gaits, such as shuffling, festinating, or freeze of gait in PD patients, by examining patterns of various gait cycle parameters and other possible characteristics in acceleration patterns. Ambulatory rehabilitation, gait assessment and personal telecare for people with gait disorders are also possible applications.

## 2. Method

### 2.1. Instrumentation

**Figure 1.**
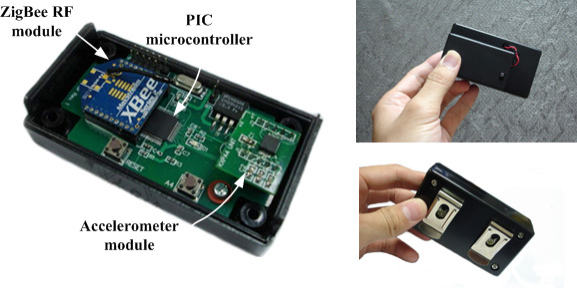
The prototype of the wearable motion detector.

The wearable motion detector is a single waist-mounted device that measures trunk accelerations of human movements. Figure 1 shows the circuit board assembly and the prototype of the wearable motion detector. It uses a tri-axial accelerometer module (KXPA4-2050, Kionix) that senses accelerations in the sensitivity of 660 mV/g over the selected range of ±2 g. The accelerometer module has an internal built-in low pass filter at cut-off frequency of 50 Hz. This circuit limits the bandwidth of the signal outputs and therefore reduces the higher frequency components which are not related to actual human movements. A PIC microcontroller (PIC18LF6722, Microchip) offers flash memory of 128 kbytes and SRAM of 3,936 bytes. It samples the analog output signals via a 10-bit A/D conversion at the sampling rate of 50 Hz. Real-time signal processing can be implemented in the PIC microcontroller. The wearable motion detector also uses a wireless 2.4 GHz ZigBee RF module (XBee 2.0, Digi International) to transmit real-time recognized gait cycle parameters to a personal computer (PC). The gait cycle parameters can be displayed on the PC screen for telemonitoring and data logging. Powered by 3 AAA batteries (DC4.5V), the wearable motion detector measures 90 mm × 50 mm × 25 mm in size and 120 g in weight. The battery life can last up to approximately 50 h when the wearable motion detector is continuously in use.

The wearable motion detector was clipped to the pant belt of the subjects. The detector is positioned near the middle between the anterior superior iliac spine and the right iliac crest around the pant belt. The pant belt on the subject was fastened medium to tight without causing discomfort to the subject, as this adjustment can reduce misalignment of orientation and vibration of the instrument which will generate signal artifacts and noises.

### 2.2. Subjects and Gait Data Collection

For validation purposes, five elderly Parkinson’s disease patients (four males and one female, 78 ± 9.8 yr) diagnosed as Hoehn & Yahr (H&Y) stage II to III and five young healthy subjects without mobility impairment (all males, 26 ± 3.1 yr) were recruited for gait data collection. The data collection was approved by the Institutional Review Board (IRB) at the Far-Eastern Memorial Hospital, Taiwan. The recruited subjects were provided with necessary information about the measurement and they gave their informed consents before the data collection.

Before gait data measurement, the Timed Up and Go (TUG) test, which is a validated simple and quick measure for mobility assessment [15], was conducted to quickly screen the mobility level of all the subjects. The time for the PD patients to perform the TUG test is 23.9 ± 7.9 s, while the young healthy subjects took only 10.6 ± 2.2 s. The distinct difference in the TUG test results show a generally degenerative mobility level in the PD patient group.

The 5-meter-walk test (5WMT) was conducted in a laboratory. In the 5MWT, the subjects wore the wearable motion detector at their waists while walking on a 5-meter level walkway three times at their own normal and faster walking paces. The accelerations along the vertical (VT), antero-posterior (AP) and medio-lateral (ML) directions were recorded at the sampling rate of 50 Hz. The initiation of data sampling of the wearable motion detector triggered the start of synchronized video recording during the test for gait observation and cadence validation.

### 2.3. Gait Cycle Parameters Recognition

Walking can be generally regarded as a repeated movement of human body. Therefore the measured accelerations during walking should also reveal periodic signal patterns. The autocorrelation procedure is a method to estimate the repeating characteristics over a signal sequence containing periodic patterns and irregular noises. Moe-Nilsson *et al*. have demonstrated the fundamentals of the autocorrelation procedure for computing gait cycle parameters, which is the basis of the recognition method in this study [16].

In the work by Moe-Nilsson *et al*., and the subsequent work by Yang *et al*. [17] and Keenan *et al.* [18] using autocorrelation for gait cycle analysis, the gait cycle parameters were computed in an off-line manner. In this paper, the autocorrelation procedure is implemented in an embedded wearable system for real-time gait cycle parameter recognition, which extends its possible applications. The autocorrelation procedure for computing gait cycle parameters is described below. Practical considerations and limitations for implementing the autocorrelation procedure in the embedded system for real time gait cycle parameter recognition are also discussed.

Consider a time-discrete acceleration sequence containing *N* signal points [*x*_1_,*x*_2_,*x*_3_,⋯,*x*_*N*−1_,*x_N_*], Equation (1) calculates the autocorrelation coefficient *a_m_*, which is the sum of the products of *x_i_* multiplied by another signal *x_i+m_* at the given phase shift *m*. The phase shift *m* can be either positive or negative integers from 0 to *N*−1, or from 0 to 1−*N* . Therefore, from an *N*-point acceleration sequence, its autocorrelation sequence *A* = [*a*_−*m*_,*a*_−*m*+1_,⋯,*a*_0_,*a*_1_,*a_m_*_−1_,*a_m_*] can be represented by 2*N*−1 autocorrelation coefficients obtained at every phase shift *m*. The autocorrelation sequence can either be “biased” or “unbiased”. The unbiased autocorrelation sequence as shown in Equation (2) is preferred because the biased method generates noticeable attenuation of coefficient values next to the zero phase shift from a limited number of data:

(1) $$a_{m}=\sum_{i=1}^{N-|m|}x_{i}x_{i+m}$$

(2) $$a_{m}^{\mathrm{unbiased}}=\frac{1}{N-|m|}\sum_{i=1}^{N-|m|}x_{i}x_{i+m}$$

**Figure 2.**
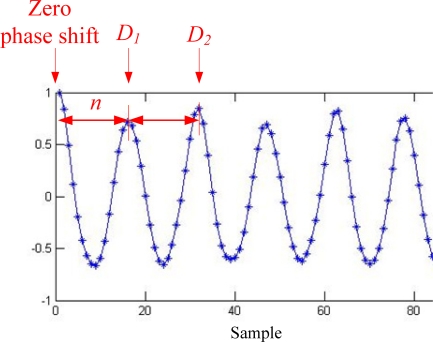
The example of an autocorrelation sequence computed from the vertical acceleration measured at waist during walking.

The segments from *a*_−*m*_ to *a*_−1_ and from *a*_1_ to *a_m_* in an autocorrelation sequence are symmetric with its zero phase shift *a*_0_ located at the center of the sequence. Normalized to 1 at the zero phase shift *a*_0_, only the right half segment *a*_0_ to *a_m_* of the autocorrelation sequence is considered for simplicity. Figure 2 depicts an example of an autocorrelation sequence computed from the VT accelerations measured at waist during normal walking paces. The first coefficient peak *D*_1_ next to the zero phase shift indicates the first dominant period, and the second peak *D*_2_ the second dominant period. The peaks *D*_1_ and *D*_2_ can be detected by a simple derivative-based method and zero-crossing identification, which are commonly used in detecting peaks in physiologic signals, such as PQST points in ECG signals. The two peaks can also be found on the autocorrelation sequence computed from the AP acceleration sequence. From repeated observations, the two peaks on the VT autocorrelation sequence appear in the same positions as the peaks on the AP autocorrelation sequence. Therefore superimposing the VT and AP autocorrelation sequences can better highlight the exact positions of the two peaks.

The following gait cycle parameters can be derived from the autocorrelation sequence:

**Step regularity and stride regularity:** A signal sequence with perfectly repetitive pattern produces its autocorrelation sequence containing the peak magnitudes identical to its zero phase shift at every dominant period, *i.e*., the magnitudes at every dominant period is 1 for a normalized autocorrelation sequence. The magnitude *D*_1_ represents step regularity, as defined by Moe-Nilssen *et al*. [16]. This is because the first dominant period indicates the maximal similarity between the acceleration sequence and its *m*-point shifted duplicate. The *m*-point span approximates the duration of a step. Similarly, the second dominant period indicates the maximal similarity between the acceleration sequence and its 2-step shifted sequence, and therefore the magnitude *D*_2_ represents stride regularity [16]. Note that the first and second dominant periods do not represent which of the steps (left-leg or right-leg) as there is no such information given in the autocorrelation procedure.

**Step symmetry:** Step symmetry is defined as the ratio of step regularity to stride regularity, or *D*_1_ / *D*_2_ that indicates the symmetry between two steps of both legs [16]. In this paper, the step symmetry is *D*_1_ / *D*_2_ if *D*_2_ ≥ *D*_1_, and *D*_2_ / *D*_1_ when *D*_2_ < *D*_1_. Note that this definition is altered from the definition originally given by Moe-Nilssen *et al.* [16] and its modified version by Yang *et al.* [17]. In this definition, the step symmetry always ranges from 0 to 1, which would be more interpretable.

**Cadence:** Cadence is the step rate per minute. Let *S* be the number of steps taken over the time period *t* (in second). Cadence can thus be expressed as Equation (3). The number of steps *S* can be expressed as the number of the total samples *N* divided by the number of the coefficients *n* between the zero phase shift and the first dominant period, *i.e*., *S* = *N* / *n*. The time period *t* during walking can also be alternatively expressed as *N* divided by the sampling frequency *f*, *i.e.*, *t* = *N* / *f* . As a result, cadence (*c*) can be estimated by Equation (4), which was given by Moe-Nilssen *et al.* [16]:

(3) $$c=60\frac{S}{t}$$

(4) $$c=60\frac{S}{t}=60\frac{\frac{N}{n}}{\frac{N}{f}}=60\frac{f}{n}$$

A non-overlapping sliding window technique is used to cyclically produce gait cycle parameters in real time from the VT, AP, and ML accelerations. Longer window lengths can produce more precise gait cycle parameters because the data of more steps is included. However, considering the real-time processing constraints, longer window lengths will cause longer computation latency and less reserved margin of memory capacity. Taking the hardware memory capacity and computation latency of the wearable motion detector into account, the window length is set at constant 3.5 s. According to the assumed average cadence of 105 steps/min that approximates regular walking cadence 75–135 steps/min [19,20], the choice of 3.5-second data can includes approximately 6 steps, which should be sufficient for autocorrelation processing.

## 3. Results and Discussion

### 3.1. Results of the Experiment

**Figure 3.**
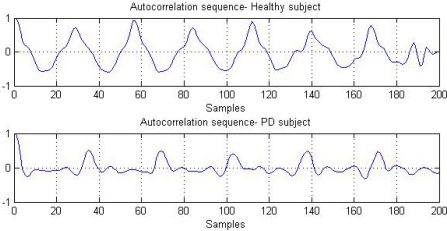
The example of autocorrelation sequences (VT acceleration) computed from a young healthy subject (above) and a PD patient (below).

To derive the gait cycle parameters, the VT acceleration was first used to compute the autocorrelation sequence because the VT component can represent the characteristics of each step for classification of walking [21]. In this study, the autocorrelation sequences computed from the healthy young subjects exhibit a more smooth and monotonic pattern, while the counterparts obtained from the PD patients contain visible fluctuations and are less regular. Figure 3 shows one example of such observations in this study. The peak magnitudes at the dominant periods from a PD patient’s pattern are relatively lower than that from a healthy young subject’s pattern.

**Figure 4.**
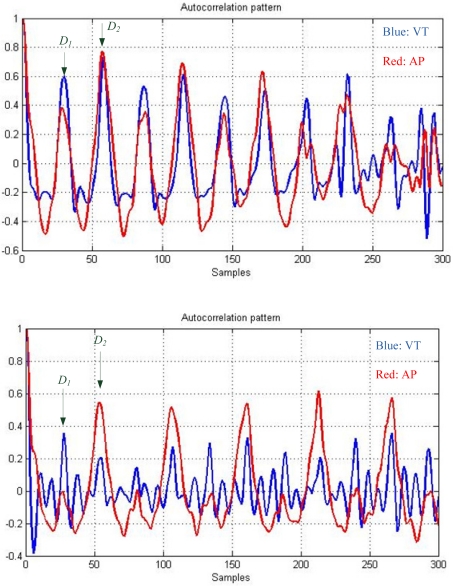
The example of the VT and AP autocorrelation sequences computed from a young healthy subject (above) and a PD patient (below).

Figure 4 shows the autocorrelation sequences computed from VT and AP accelerations obtained from a healthy subject and a PD patient. The young healthy subject has better step and stride regularity (larger magnitudes at the peaks *D_1_* and *D_2_*) than the PD patient. This shows that the periodic characteristics of gait in the PD patient are not steadily and perfectly reproduced. It is shown that the dominant periods on the VT and AP patterns coincide with each other, even though the two patterns may vary differently. The comparison of both the VT and AP autocorrelation sequences can improve the accuracy in identifying the dominant periods when the dominant periods cannot be clearly determined from the VT autocorrelation sequence alone.

Table 1 shows the mean values of the recognized gait cycle parameters of all subjects of the two groups. Comparing the cadences derived from the autocorrelation procedure, the average cadence of the PD group (102.2 ± 15.2 steps/min) is slightly higher than that of the healthy group (98.6 ± 5.8 steps/min). The elevated cadences at longer TUG time for the PD patient group indicate reduced step length and walking speed, which conforms to the literature result [1]. In fast 5MWT, the average cadence of the PD group was 108.1 ± 15.6 steps/min, which was approximately 5.8% increased from their normal cadences. The healthy group had an average cadence of 113.9 ± 6.2 steps/min in fast 5MWT, which was approximately 16.9% increased from their normal cadence. This indicates a limited performance margin for the PD group due to their degenerative mobility.

For the gait regularity and symmetry, the PD patient group has the step regularity of 0.39 ± 0.16 and the stride regularity of 0.43 ± 0.2 during normal walking paces. The healthy group has higher step regularity (0.61 ± 0.14) and stride regularity (0.79 ± 0.09). Similar trends can be observed in their fast walking paces. Therefore, the result shows that the PD patients cannot well regulate their repeating steps and strides compared with the healthy group. Note that the step symmetry during normal walking from the PD group is slightly higher than that from the healthy group, while the step symmetry during fast walking in the PD group is lower than that in the healthy group. This mixed results regarding step symmetry need further investigations. In the current definition of step symmetry, it is possible to obtain higher step symmetry from the gaits with both low step regularity and stride regularity that are close to each other. Altered definitions of step symmetry have been used [18], and it is also of important interest in the future to investigate which definition can be suitably applicable.

The cadence obtained by the method was compared with that measured from the synchronized video. The mean absolute percentage error is 4.89%. As the subjects repeated each test item three times during the data collection, it is hypothesized that the subjects repeated the tests without significant variance so that the results of gait cycle parameter recognition can be valid. Therefore, the one-way ANOVA test was used to investigate whether each test item shows significant variance in both the subject group. In general, there is no significant difference (significant level 0.05) between the results from each test of the subjects. Note that because some of the PD subjects were unable to perform fast 5MWT, the test in the PD subjects’ fast 5MWT are excluded in Table 1 due to the limited data samples.

### 3.2. Discussion

Several studies have reported the use of the vertical accelerations for autocorrelation procedures [16,17]. In this study the VT, AP and ML acceleration components were compared to examine which axis is most sensitive to steps and produces identifiable pattern related to the gait cycle parameters. From our observation from the autocorrelation sequences of the 10 test subjects, the ML acceleration component is considered least descriptive and least sensitive to walking movement. This was also observed in a previous study by the authors [22]. In the study by Keenan *et al*. [18], the VT and AP components were also used for autocorrelation process for the same reason even triaxial accelerations were measured. Though the ML acceleration component measured from the back over the L3 region has been shown good results in autocorrelation procedure [16], this could be because of different positions to attach the devices were used. In the future developments, the ML accelerations may be used to distinguish left-leg or right-leg steps [23]. The real-time 3D orientation of the trunk can be synchronously computed from the vertical acceleration, or from the combination of the three acceleration components for better accuracy. However, trunk orientation reveals less information in interpreting gait patterns, and thus it is not used throughout the recognition procedure.

The wearable motion detector developed in this study measures walking movement at the sampling rate of 50 Hz. As a low-pass filter circuit (*fc* = 50 Hz) was internally built in the accelerometer module to reduce the signal components of higher frequencies that would not be relevant to human movements in daily living, ideally the sampling frequency above 100 Hz should be better. However, the sampling rate 50 Hz here is used because of the limitations of available memory capacity (data to be buffered), computation capability of the low-cost PIC microcontroller, and the constraint of a real-time system requiring minimized cyclic processing time. Moreover, intense and faster movements could rarely occur in daily living home environments. Compounding the above considerations, the sampling rate 50Hz was used, though higher sampling rate is certainly better if capable hardware is available.

Step regularity, stride regularity and step symmetry obtained from the autocorrelation procedure are the general temporal gait estimates in terms of signal periodic characteristics. Cadence can be easily validated by comparing the synchronized video recording. Tura *et al*. have validated the step and stride regularities to well correlate with the indices obtained from step and stride duration measured from pressure insoles [24]. Offline analysis using autocorrelation method usually processes large acceleration data to compute the gait cycle parameters that indicate its overall gait performances. Real-time recognition of the gait cycle parameters using cyclic processing may be affected by a high instantaneous variability. An extra continuous 25-meter walk data from three healthy young subjects in their constant walking paces was additionally used to check the effect of using shorter window lengths to calculate the gait cycle parameters. As shown in Table 2, the gait cycle parameters obtained from the multiple sliding windows (window length 3.5 s) can moderately to highly approximate the gait cycle parameters obtained from the entire single window. However, for data in varied walking speeds, it was found that gait cycle parameters obtained from the multiple sliding windows were sensitive and can reflect the episodic signal variability.

**Figure 5.**
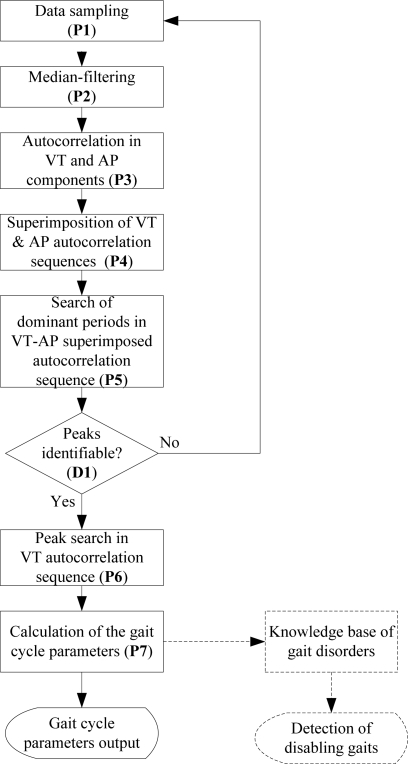
The process flowchart of real-time gait cycle parameters recognition.

As real-time gait cycle parameter recognition is developed, continuous detection of disabling gaits could be applicable. Figure 5 shows the process flowchart of the algorithm. After the data sampling (P1), the median-filtering process (P2) is applied to the sampled data to eliminate signal spikes that are not related to human movement. The VT and AP autocorrelation sequences are generated (P3) from the sampled data. In the process P4 both the VT and AP autocorrelation sequences are superimposed to obtain a peak-highlighted sequence which is used for peak detection. The P5 process locates the positions of the first and second dominant periods in the VT-AP superimposed sequence. If the process D1 fails to identify the positions of the first and second dominant periods in the VT-AP superimposed sequence (D1 = No), the procedure returns to the process P1 to restart next data sampling. If that positions are identifiable (D1 = Yes), the peak search process (P6) finds the first and second dominant periods, *D_1_*, and *D_2_* in the VT autocorrelation sequence according to the peak position located from the VT-AP superimposed sequence. The gait cycle parameters are then computed in the process P7 and the estimates output is provided prior to the next new processes from P1.

With the available gait cycle parameters in consecutive identification periods, a knowledge base of gait disorders and the pattern characteristics, *i.e.*, shuffling, festinating and freeze of gaits can be integrated in the future to facilitate the potential capability in real-time and continuous detection of disabling gaits in PD patients.

## 4. Conclusions

This paper presents the use of a wearable motion detector for real-time gait cycle parameter recognition. The wearable motion detector is a single waist-mounted device that utilizes a tri-axial accelerometer to measure trunk accelerations during walking. The autocorrelation procedure is used to estimate cadence, step regularity, stride regularity and step symmetry from the measured trunk accelerations in real time. In this study, the gait cycle parameters among the two subject groups of different mobility (PD patients and the young healthy adults) can be quantified and distinguished by the system. The wearable motion detector has been developed by the authors for real-time physical activity identification, and its connection to a telecare system has also been presented [22]. The system developed in this paper can lead to future research interests and development regarding real-time detection of gaits disorders in PD patients. Mobility assessment, ambulatory rehabilitation for PD patients can be the possible applications.

## Figures and Tables

**Table 1.** Gait cycle parameters derived from the subjects.

		**PD**		**Healthy**	
		**Mean ± SD**	***p*-value**	**mean ± SD**	***p*-value**
**TUG test**		23.9 ± 7.9 s	0.852	10.6 ± 2.2 s	0.982
**5MWT (normal)**	**Cadence**	102.2 ± 15.2	0.835	98.6 ± 5.8	0.980
	**Step regularity**	0.39 ± 0.16	0.952	0.63 ± 0.13	0.856
	**Stride regularity**	0.43 ± 0.20	0.934	0.80 ± 0.09	0.066
	**Step symmetry**	0.81 ± 0.14	0.575	0.78 ± 0.16	0.901
**5MWT (fast)**	**Cadence**	108.1 ± 15.6	n/a	113.9 ± 6.2	0.533
	**Step regularity**	0.37 ± 0.17	n/a	0.76 ± 0.08	0.545
	**Stride regularity**	0.47 ± 0.12	n/a	0.80 ± 0.08	0.360
	**step symmetry**	0.75 ± 0.22	n/a	088 ± 0.09	0.924

**Table 2.** Gait cycle parameters derived from multiple sliding windows and the entire single window.

	**Multiple sliding Windows**			**Entire single window**
	**Mean coefficient of variance (CV)**	**Mean percentage error**	**Mean ± SD**	
**Cadence (steps/min)**	1.21%	0.67%	113.3 ± 4.1	111.1
			103.4 ± 0.0	103.4
			111.1 ± 0.0	111.1
**Step regularity**	8.53%	2.44%	0.763 ± 0.073	0.793
			0.679 ± 0.042	0.665
			0.785 ± 0.077	0.773
**Stride regularity**	9.34%	4.47%	0.869 ± 0.128	0.903
			0.793 ± 0.052	0.746
			0.816 ± 0.061	0.877
**Step symmetry**	7.78%	2.04%	0.884 ± 0.061	0.878
			0.858 ± 0.057	0.892
			0.867 ± 0.085	0.881
